# Tuberculosis in Men and Animals

**Published:** 1882-07

**Authors:** Thomas E. Satterthwaite


THE JOURNAL

OF

COMPARATIVE MEDICINE AND SURGERY.

VOL III. JULY, 1882. No. 3.

### ORIGINAL COMMUNICATIONS.

### ART. XIV.—TUBERCULOSIS IN MEN AND ANIMALS: A STUDY IN COMPARATIVE PATHOLOGY.

BY THOMAS E. SATTERTHWAITE, M.D.

II.

IN the year 1867, while Villemin was engaged in the study of tuberculosis, but before he had published his volume, Dr. Petroff, of Kasan, in Russia, made an interesting communication bearing on the infective property of tubercle. It appears that a medical student was admitted into his hospital ward, and a diagnosis of cheesy pneumonia was made, probably in conformity with the notion that it is identical with catarrhal phthisis. The patient died, and at the post mortem examination, cavities were found in the lungs, with miliary tubercles about them, while the lung tissues intermediate between the tubercles had undergone more or less cheesy change. The disease had also attacked the liver, bronchial and mesenteric glands, and Peyer's patches. Desirous of testing the infective qualities of these lesions, Dr. Petroff* took some morsels from the cheesy deposits, and, after triturating them thoroughly in distilled water, injected into the pleural cavities of a guinea pig some of the questionable material. The animal died after

* *Petroff.* Virchow's Archiv., 44, p, 129. 1868.

a lapse of twenty days, and *post mortem* examination disclosed that the pleuræ and exocardium were rough and injected, while the lungs contained miliary tubercles.

A little later in the following year, Klebs * came to the assistance of Villemin, and offered further evidence favoring his theory that tubercle is the product of a special infection. Experiments on animals had resulted in causing an eruption of tubercles in very many instances, and the minute structure of the granules was held by him to be different in quality from those produced in the same situations by the injection of ordinary inorganic substances (quicksilver, cinnabar, etc.). He therefore placed himself in opposition to the later conclusions of Lebert † and Wyss ‡ and those of Waldenburg. § The latter observer had already shown|| that the so-called artificial miliary tuberculosis could be produced by various matters—even bits of alcoholic specimens, boiled tissues, and pulverized aniline dyes.

Langhans ¶ now contributed a small item to the increasing fund of information. He found by experimenting on rabbits that a serious error had probably been committed by Villemin, which lay in the fact that he had not "controlled" his experiments—in other words, he had not studied the condition of other like animals under a similar restraint. The re ult of this simple but very natural observation discovered to him that rabbits often have caseous deposits in their livers, which may be due to the encysted ova of strongyles ( pecies of round worms that are frequently found in our native hog, and are commonly known as lung worms), perhaps to the calcified remains of other parasites in their mature or larval forms. **

In the same year Virchow helped to throw some light on the matter, for he indicated that scrofulosis, by which he seems to have meant, at that time, caseation of the lymphatic glands, was in some way closely related to tubercle, the former disease in some way afford-

---

* *Klebs.* Virchow's Archives, 44, p. 242. 1868.

† *Lebert.* Gaz. Med. de Paris, No. – , p. 127. 1868.

‡ *Lebert and Wyss* Virchow's Arch., 40, p. 532. 1867.

§ *Waldenburg.* Be l. Klin. Woch., 51 and 52 1867.

|| *Waldenburg.* Loc cit.

¶ *Langhans.* Die Uebertragung der Tuberculose auf Kaninchen.

** The writer's experience in animal experimentation goes to show that the ova of parasites are frequently found in the livers of rabbits, while calcified ech nococcus cysts are by no means rare, especially in such animals as are bred in the cities, and fed on slops.

ing favorable conditions for the development of tubercle, but he hesitated to affirm that there was any causal relation between the two, because in some instances of tuberculosis no caseation of glands could be found after the most diligent search. They therefore merited the term *idiopathic*.

In the following year Gerlach,* a Professor in the Veterinary College of Berlin, announced certain doctrines which, emanating from so distinguished a source, received very favorable consideration. He had inoculated horses, horned cattle, goats, sheep, hogs, dogs, and rabbits with phthisical matter from men and monkeys, having also "controlled" his experiments by using other substances of various kinds. In the one series of experiments, miliary tubercles occurred very frequently, chiefly or certainly in rabbits; in the other series, where non-tuberculous matter was inoculated, no eruption of tubercles was observed. It will be noticed that now we come to an opposition of recorded facts, Gerlach claiming that non-tuberculous matter will not produce miliary tuberculosis, and Waldenburg claiming with equal positiveness that it may; both of these well-known men basing their conclusions on personal experiments.

The disease, according to Gerlach, was certainly transmitted to rabbits, and as it arose through a traumatism, spreading centrifugally, it should be called infective, as Villemin has claimed. Still the production of the artificial disease was accomplished through the caseation of the lymphatic glands in the neighborhood of the wound, and it was from and through them that the eruption ensued. Two main objections are now, very naturally, raised against these conclusions, and they are: First, that rabbits† are not adapted for advancing the settlement of the tuberculosis question, not only for reasons already named, but because there is excellent ground‡ for believing that any continued inflammatory process is likely to set up caseation, when an organic substance, whether in substance or solution, is inoculated beneath the skin. A very important and practical matter that Gerlach also laid emphasis upon, was the assertion that the ingestion of either meat or milk from tuberculous cows will develop tuberculosis in the human kind. He accordingly advocated stringent police sur-

* *Gerlach.* Virchow's Archiv., 51, p 290, 1870.

† The same objections apply to guinea-pigs.

‡ *Curtis and Satterthwaite.* Report of Investigations as to the Pathogeny of Diphtheria, p. 4, et seq., Bd. of Health Report, N. Y., 1877.

veillance over establishments where cattle were slaughtered, or where cows were kept for milking purposes.

But it was felt that these statements were in need of further consideration, and Orth,* a well-known pathologist, a pupil of Virchow and Rindfleisch, undertook an elaborate series of experiments to throw more light on the matter. It was even held to be problematical whether tuberculosis of the human family is the same disease as bovine tuberculosis (*perlsucht*, *pommeliere*), as claimed by Gerlach; for although so maintained by those who had enjoyed ample opportunity for its study (Colin † and others), both in Germany and France, it was also strongly urged, on the other hand, that both the physical characters of the lesions and the clinical history were different. To determine this matter, Orth selected a number of rabbits that gave the usual appearances of sound health. In this way he hoped to escape the danger of having accidentally selected tuberculous animals. Fifteen of them were fed with pearl matter from the lungs of tuberculous cows. On killing the rabbits subsequently, he found generalized miliary tuberculosis. Other rabbits were fed on human tubercle, but the results were negative. Positive results affected the lungs chiefly, and the types were either the gray or yellow granulations of Laennec. In some instances, cavities were found. These conclusions seem now to us rather remarkable, for in the first place tuberculosis, if it occurred, should most naturally have attacked the alimentary tract by preference, since it was by this channel that the diseased substance entered the system; and secondly, it appears strange that the human tubercle, which is conceded to be as poisonous as the bovine, should have produced no result. Still, if he could have demonstrated satisfactorily that the animals were sound before experiment, and subjected to the same conditions as in the second series, the proof of infection was strong. Especially interesting was the fact that the tuberculosis of his rabbits was similar in all respects to the human, though in the horned cattle the so-called tubercles were pedunculated rather than sessile (human), and yet they had each of them at first the same pearly lustre, and each little granulum subsequently a yellow centre, which had become caseous. In the kidneys, where the nodules are usually seen to good advantage, though by no means frequently, Orth found that the microscopic

* *Orth.* Volkmann's Sammlung, 64, 1873.

† *Colin.* Aufrecht in Schmidt's Jahrb., 144, p. 223, 1869.

appearances were the same precisely as those observed in the human family. There was the same tendency to organization of the separate granula, while injections of Berlin blue demons rated that the little vessels of the tubercle were not pervious. Some of the granulations had a reticulated structure, and the small rounded corpuscular elements, but without giant-cells; others had giant-cells. The disease also attacked the liver, spleen, mucous surfaces and omentum. The debated question as to the infective quality of the virus seemed, therefore, to be more firmly established than ever, and Gerlach's conclusions upon this subject were confirmed.

Still later, Prof. Schueppel,* of Tuebingen, reported a series of experiments from which it was concluded that "perlsucht" is the same histologically, and in its life-history, as human tuberculosis.

In the confusion of ideas which had now arisen from the fact that there was a difference of opinion as to the histological constitution of a miliary tubercle, it became essential to restudy the matter, and determine whether, by a new method of animal experimentation, the truth of the matter might be reached. Accordingly, Schottelius † performed a new series of experiments in close imitation of what was presumed to be the natural method of contracting the disease, i.e., through the air passages. He used dogs and rabbits, the latter being, as we have already shown, peculiarly susceptible to tubercle. The first step in his procedure consisted in performing tracheotomy, which he found exerted no unfavorable effect on the lungs. Next he instituted controlling experiments, to determine the effect of such foreign substances as pulverized coal, cinnabar, precipitated Berlin blue, dried and pulverized pus from a psoas abscess, fæcal matter and mould fungi, when lodged in the remotest divisions of the air passages. He concluded from these studies that ordinary *mechanical irritants* had no unfavorable influence on the lungs, at least in the way of producing tuberculosis. In fact, he maintained that even organic substances which do not suffer physical deterioration may be introduced into the lungs in large quantities and yet cause no profound disturbance. On the other hand, he found that a solution of organic substances that undergo decomposition invariably initiates processes that may bear a strong resemblance to those that are called phthisical. It is to be observed

---

* *Schueppel.* Virchow's Archiv., 56, p. 38, 1872

† *Schottelius* Virchow's Archiv., 73, p. 524. 1878.

that Schottelius* was careful in his phraseology, and the reasons for his caution became apparent when in a subsequent communication, he plainly insisted that phthisical granulations (miliary tubercles) might be mistaken either (1) for minute capillary pneumonias, or (2) for bodies caught in the lymphatic system between the alveoli, or (3) for occluded bronchioles.

Investigations in this new department of pathological research were now actively prosecuted, and Tappeiner, of Meran, who had been for some years associated with this movement, published his conclusions in 1879,† embodying work done under the supervision of Prof. Von Buhl, of Munich. Taking dogs as his subjects, he subjected them to an atmosphere laden with phthisical sputum in a state of minute division, and forced them to inhale it constantly from twenty-five to fifty-one days. At post-mortem examination they were invariably found to have miliary tuberculosis, chiefly in the lungs, but also in other organs.

Schottelius‡ finally repeated Tappeiner's experiments, but forced the animals (also dogs), to respire an atmosphere laden with various organic substances in a state of fine division (bronchitic sputum, Limburger cheese, powdered brain), and his results in this series were precisely the same as at first. These two observers, therefore, claimed diametrically different results in two series of experiments that seemed to be practically alike. As further opposed to Tappeiner, the experiments of Wargunin, though of recent date, may be noted.§ Acting under the direction of Prof. Rajewski, he took sixteen dogs and submitted them to the following tests: Some were forced to inhale a vapor which contained phthisical sputa; others, on the other hand, inhaled a similar vapor, but laden either with emphysematous sputa, meal, Schweizer käse, or disinfected tuberculous sputa. The result was always the same in quality, the findings varying only in grade, which bore a direct relation to the duration of time between the date of the experiment and the killing of the animal.

In each that was examined the pleuræ and lungs contained firm translucent tubercles, that measured about two millim. (1-15 inch) in breadth. The mucous membrane of the larynx and bronchi was

---

* *Schottelius.* Virchow's Archiv., 73, p. 574. 1878.

† *Tappeiner.* Virchow's Archiv., 74, p. 397. 1879.

‡ *Schottelius.* Med. Central, b'. 3. 1878.

§ *Vratsch.* No. 6, All. Med. Central bl. Apr. 8, 1882.

intact. Microscopic examination also showed that the process began in the finest bronchioles, and then spread to the adjacent alveoli. This secondary process was catarrhal in its nature, and, extending to the bronchioles, occluded them with granulation tissue, that ultimately tended to the development of connective substance. This process, therefore suggested the desquamative pneumonia of Buhl. The tubercles were devoid of vessels and tended to central caseation. The whole process resembling that of tuberculosis, should, he thinks, not be mistaken for tubercular phthisis, but be accredited to mechanical irritation. Recovery ensued, as was shown by several post-mortem examinations, after a lapse of some months.

The later conclusions of Tappeiner appear to define more closely his views on the infectiousness of the disease. Thus repeated experiments on dogs, in which they were fed with tuberculous sputa, failed to develop lesions of the intestinal tract. He believes, therefore, that the disease does not enter in this way, nor does he think that the ordinary liquid sputum of phthisical persons conveys the disease, as experiments on rabbits showed. But being convinced that it is contagious, he thinks it is inhaled in the dry dust of rooms. (*Deutsch Archiv. of Klin. Med.*, *29*. *All. Med. Central Zeitung.*)

The views of Rindfleisch,* which are appropriately introduced at this point, give some sort of an explanation of these seeming differences, and they are worthy of record because his teachings and writings have introduced him to a large and interested circle, both abroad and in this country. He finds three varieties of miliary tubercle, if I understand him correctly.

One is the peculiar tubercle that occurs in the artificial disease of guinea-pigs, the second is the fibroid of syphilis, and the third is the lymphadenoid, notably of scrofulous persons, and containing the peculiar epitheloid bodies known as "Schueppel's corpuscles." The term "miliary" he rejects, for the reason that they are not always or generally the size of a millet seed, but often as large as a pea, or even larger. They are made up of minute spherules (submiliary bodies), which have a diameter varying between one-fifth and one-seventh millim. (1-150th to 1-210th of an inch). The epitheloid bodies (Schueppel's) are the swollen and granular connective tissue corpuscles of the connective tissue trabeculæ, and the bloodlessness of the gran-

---

* *Rindfleisch.* Ziemmsen's Cyclopæd., Am. ed., v. p. 642.

ulum is due to the involvement and obliteration of the blood-vessels or lymphatics. In the subsequent retrograde metamorphosis that these nodules undergo, centres of infection are developed which poison the adjacent tissue and give rise to other granules similar to themselves in character.

This explains how tubercularization may spread from caseous centres centrifugally into the parts immediately surrounding them, but fails to account for systemic infection. Ponfick had noted a single instance where ulceration into the thoracic duct had taken place, but such an accident would naturally be rare. Weigert,* however, has suggested another way, and cited three cases of his own to prove it. In these, ulceration and perforation of a pulmonary vein took place, so that it became comprehensible how the infecting matter might be carried to the left side of the heart and then distributed through the arterial system to any part of the body.

According to Rindfleisch,† the initial tubercle is placed in the walls of the very smallest bronchiole, just where it enters the vesicle, and, of course, in the connective tissue septum. But to understand the matter thoroughly, it is well to study the minute structure of the human lung, which is a very complex affair as compared with this organ in many of the lower animals. In the first place, it is made up of lobules, bound firmly together by connective tissue. These lobules contain from two to twenty acini or lobulettes‡ (Waters), each supplied with a terminal bronchus or bronchiole, which divides into short canals, the alveolar passages, three in number for each acinus. The secondary branches which the alveolar passages give off are called infundibula. Both the infundibula and the alveolar passages and their termini are ballooned out with little sacs closely disposed side by side and known as alveoli or air vesicles. The first lesion in pulmonary tuberculosis consists in a nodular infiltration of every angle and point, where the bronchiole meets the acinus. Each nodule increasing in size, they gradually near one another, and finally meeting, constitute by their agglomeration miliary tubercles, and the internal conformation of each varies according to the number and size of the submiliary bodies of which they are composed.

Before closing this part of our subject it is well to notice a plan

* *Weigert.* Virchow's Arch v., 77, p. 269. 1879.

† *Loc. Cit.*

‡ *Westbrook* Satterthwaite's Manual of Histology. 1881.

that has been suggested recently by Martin* to prove that an eruption is *true* rather than *false*. He claims that, the microscope having failed, he can determine it by *serial inoculation*. While the artificial or false tubercle will die out in one generation, the true will continue in vigor and even increase in intensity by successive inoculations. A true tubercle will produce generalizations of the disease—a false will not.

In this connection also a very interesting and suggestive case has been published by Dr. J. R. Wolfe,† in which, following a blow, a little tumor formed in the upper segment of the iris. It was removed by operation, but the wound did not heal kindly, and finally the eye was enucleated. On microscopic examination, the iris and ciliary body were found studded with tubercles, showing in their centres giant cells. In this instance, the tubercles followed a blow, and the child was subsequently found to be cachectic, while indolent, ichorrous ulcers developing on both legs. The teachings of this incident would seem to be, that the traumatism developed the tubercles, and that it subsequently became generalized; or, possibly, that the condition of the patient determined the inflammation to be tubercular. In either case, the traumatism seems to have been the exciting cause.

Having thus enumerated the principal views that have been held during the past ten years, by those who have devoted their attention to tuberculosis from the standpoint of pathological histology, it will not be amiss to conclude with those of Ziegler,‡ whose excellent pathological manual is a model of judicious personal work.

Tubercle (miliary) he holds to be a non-vascular cellular nodule that develops until it attains a certain size, and undergoes caseation. Usually giant cells are found in the centre of each granulum, and their nuclei are either disposed peripherically in the corpuscle, or at one of its extremities. The mononuclear elements are in part lymphoid corpuscles, in part endothelial or epithelial bodies (Schueppel's epitheloid corpuscles). These two latter varieties are coarsely granular as compared with the finely granular lymphoid bodies. Now, these giant cells and epotheloid corpuscles may, according to Ziegler, be found in ordinary granulation tissue, or as the result of the coalescence of epithelial bodies, but neither they nor any other textural elements are charac-

---

* *Martin.* Rev. de Med., No. 4, p. 290. 1882.

† *Wolfe.* Brit. Med. Jl., Mar. 4, 1882.

‡ *Ziegler.* Lehrb. d. allg. u. spec. Path. Anat., I. p. 156 et seq.

teristic of tubercle. In fact, adjacent tubercles will exhibit one or other of these peculiar bodies or no one of them. They are, however, specially prone to exist in tubercular neoplasms, and constitute features that peculiarly distinguish them. Yet precisely the same appearances may be found in artificial tuberculosis produced by mechanical irritants, or in the nodules (tubercles) of Lupus.

And still in these two affections the clinical phenomena are so very distinct and different, that from a practical point of view, the histological similarity of the lesions leads to no confusion. It is important, however, to note this resemblance, which offers corroborative proof that there is nothing distinctive in the minute structure of the granulum. If, then, we regard this miliary body as the product of inflammation, we may very naturally connect it with other inflammatory phenomena, and as such it has long been classed by the laity. But what causes this peculiar phase of inflammation, and why are certain individuals selected? Unfortunately, we seem to be far from having the means of settling the question. Cohnheim desponded of ever obtaining any satisfaction in the inquiry. Rindfleisch referred it to a scrofulous tendency, while Koch* and others have proclaimed that the agent is a microphyte.

It is well to remember, however, that phthisis is governed by certain laws that are generally recognized and have been deduced from the most elaborate series of statistics. For instance, it is known that it usually attacks certain families by preference, selecting those members that have not been favored by the best physical development, or whose nutrition has been retarded by harmful conditions, such as bad air or food; that it elects certain periods of life for its visitation, the first most dangerous period being between the fourth and seventh year of life, and the second between the fifteenth and twenty-fifth, while after thirty-five the susceptibility diminishes; it has a

* In a recent number of the *Berl. Klin Woch* (No. 15, 1882) Robert Koch, whose name has for some years back been associated with the study of infective diseases, chiefly in relation to their etiology, has published a full description of the parasite that he finds associated with tubercle. He has performed a very large number of experiments, employed new dyes to differentiate the bacteria, and has propagated them (according to his description) by methods that appear calculated to produce accurate results. It is too early now to decide as to the value of the experiments, though it is proper to say the writer has not as yet been able to verify his conclusions, in some preliminary experiments that have been made.

marked tendency to break out in scrofulous* persons, and occur as a sequel to the bronchitis of measles or whooping cough and the pneumonia of diabetes mellitus.

But why do not ordinary attacks of pneumonia or bronchitis produce it? If, as Rindfleisch claims, it comes from a caseous centre such as the inspissated pus from a catarrh in the apex, how does it happen that these products often lie dormant in the body, and yet no tuberculosis results. To one who has made many post-mortem examinations this is notoriously a fact. What, then, is the agent that determines this special train of symptoms? Is the cause to be found inside or outside of the body?

Now, it is a rule, in such inflammations, that blood-vessels are not developed, so that there is no effort made toward restoration of the part, but rather towards its destruction, through the coalescence of the cellular elements and the infiltrated tissue, which together combine to form a cheesy *focus*.

We have seen in the preceding account of tubercle and its allied manifestations, in the human subject, that our modern idea of pulmonary, laryngeal and renal phthisis relegate the fundamental lesions to tubercle, of which the first chronological factor is the gray granulation of Laennec or of Bayle, which has as a striking peculiarity, a central caseation. Accordingly adenoid tissue, which at one time was held to represent the anatomical structure of tubercle, has ceased to have its former prominence, while the giant cells and Schueppel's epitheloid corpuscles have equally with the former failed to indicate pathognomonic formations, and this simply because the later revelations of the microscope have taught that giant cells are found in a variety of other situations, and because the epitheloid corpuscles may indicate merely the swollen or otherwise altered conditions of the ordinary connective tissue corpuscle, less probably the swollen leucocytes (Cohnheim).†

---

* Our modern definition of scrofula dates from the descriptions of Virchow and Rindfleisch, and indicates a habit of the body in which there is a peculiar variety of inflammation. The localities most affected are the mucous membranes and the lymphatic glands, although the bones, joints and skin are often selected. Such inflammations are mainly characterized by their protracted duration and a local infiltration, with a tendency to fatty metamorphosis and death of the part through the medium of a caseous change. The connective tissue elements cause the infiltration, by swelling to an unnatural size and becoming fatty

† *Cohnheim.* Aufrecht in Schmidt's Jahrb., 184, p. 291.

And now we come to the question of pearl disease and the tuberculosis of animals and their relations to human pulmonary consumption. First of all, these animals are not equally susceptible to tubercle. Some, such as dogs and cats more especially, have no tendency to it. Crisp,* who has enjoyed unusual facilities in collecting data on this point, says that he has met with tubercle in more than one hundred different specimens of animals, quadrupeds, birds and reptiles. On the other hand, he has never seen the disease among invertebrates, but in fishes has seen fibrous tumors showing caseation at their centers. In reptiles, especially the orchidians, he has frequently found tubercles. In birds, such as the domestic poultry, he had found collections of cells about the smaller branches of the portal vein and about the malpighian corpuscles of the spleen. They softened and finally became cretaceous, and when removed from the body had a stony hardness. In some birds they resembled the disease in quadrupeds. In mammals, especially the quadrumana, he found that the disease resembled the human variety, though the livers and spleens were more often attacked than the lungs. But vegetable-feeders were most prone to phthisis. Captivity, also, has undoubtedly some important influence on all animals, as menageries and zoological gardens show. Fleming quotes Cruzel, who states that in 100 old and fattened oxen fully one-half were found, when slaughtered, to be tuberculous, while in cows the proportion that were unaffected was very small. So, according to Lafosse, Professor in the Toulouse School, animals used for agriculture or for breeding escape the disease, as also those who have to yield milk for a short time only, while, on the other hand, excessive and long continued lactation promotes it. But probably the proportion of tuberculous animals varies much with the locality, for, according to Creighton, of oxen slaughtered in Augsburg in 1877, only 2.6 per cent., and of cows only 4.75 per cent. had tubercles. The peculiarities of this disease, which most observers declare to be identical with the human tuberculosis, are that it is deposited in enormous massess throughout the organism, including not only the lungs, but the liver, spleen, kidneys and mesenteric glands. Rarely they are found in the muscles.

To determine the identity or non-identity of the two, the following anecdote is related.† On July 26th, a handsome and well-developed

* *Crisp* Trans. of the Path. Soc. of London, 1873. p 346.

†*Klebs.* Virchow's Archiv. 49, p. 29. 1870.

calf, four weeks old, was inoculated beneath the skin of the belly with human tubercular matter in solution. On October 22d following, the animal was killed, and the great omentum, with a considerable portion of the stomach, were covered with pedunculated bodies, in which there was a central calcareous mass, that had all the histological characters of the pearl bodies. Professor Chauveau, of the Lyons Veterinary School, reached a similar conclusion as to the identity of the two diseases by a different method. Having suspected that *ingested* virus was capable of producing the disease, he selected four calves, and compelled three to swallow each about thirty grammes (one ounce) of tuberculous matter from an old phthisical cow. Of these, two were killed fifty-two days later, and in one *carreau* (mesenteric phthisis) was exceedingly well marked. Some of the mesenteric glands were as large as one's fist, and the intestinal eruption was very distinct. The liver, spleen and kidneys were not affected. Gray and "crude" tubercles were numerous in the lungs and bronchial glands. The cervical and submaxillaries were also involved and ulcers were found in the larynx. The second calf was in some respects similarly affected, except that laryngeal phthisis was absent and the intestinal eruption was less pronounced. Chauveau states as the result of his experimentation, that bovine animals contract tuberculosis through the infection of *digestive indigestion*, as they may take anthrax and vaccinia, as sheep take variola, as solipeds take glanders, and as men take small-pox. He therefore holds to the view that tuberculosis is contagious. Leisering, of the Dresden Veterinary School, further sustained these views by the results of his experiments on a sheep fed for three days upon the tuberculous lymphathic glands of a cow. The sheep was killed on the fifth day, and the lungs, bronchial glands, liver and intestinal mucous membrane were found ulcerated and studded at various points with small tuberculous tumors.

Zürn, of the Jena Veterinary School, fed pigs (which are not specially prone to tubercles) first with the milk and then with the meat of a phthisical cow, and claimed that he produced various degrees of tuberculosis. According to Fleming, an old observer, Cruzel, already alluded to, noticed that there was a tendency for animals to become tuberculous when they cohabited, *i.e.*, occupied the same or adjacent quarters. Viseur, of Arras, also quoted by Fleming, noted the same thing. Zundel, of Alsace, noted this in the hardy Swiss cattle who, in association with other phthisical cattle, would succumb while others, less well cared for, but not so exposed, would remain healthy for years. Grad, a veterinary surgeon of Alsace, furnished some very satisfactory evidence pointing to the same source of the disease. During a twenty-five years' experience he had always doubted the contagious or infectious quality of tubercular virus, but supposed that it should be regarded as an hereditary disease, similar to congenital syphilis. But though he had known five animals occupying the same stall in succession to be taken with cough and die of consumption, he was inclined to think it a matter of coincidence only. However, being urged to test the point, a young and healthy cow, three years old, with no hereditary history of tubercle, was selected, and placed in the stall. After calving, a short cough set in, and in twelve months phthisis was well developed.

The proof that there was a contagious element present in that particular locality was further strengthened by the observed fact that after the stall had been thoroughly cleansed and disinfected, tuberculosis did not again occur in it. The somewhat radical views of Prof. Gerlach now deserve mention, as they gave impetus to much of the work recently done under the auspices of various continental governments. He stated, as the result of his personal experience: That tuberculosis in cattle is very infectious. That tubercles covering the serous membranes, or any organs, are as infectious and produce the same tubercles as the tuberculous matter of the lungs. That pulmonary tuberculosis in cattle is identical with that of the human family. Infection can be produced by inoculation or by ingestion of tuberculous matter. The flesh of tuberculous animals has the power of setting up tuberculosis, when ingested, though with less intensity * than when inoculated.

Fleming, after recording these views, adds there is every reason to view with grave suspicion the use of flesh from phthisical cattle, especially if the disease be much advanced and the tissues generally are involved. But with more reason the milk from cows affected with tuberculosis should be prohibited, particularly when given to infants, who mainly rely upon this kind of food for their nourishment, and whose powers of absorption are very active. Even if such milk did not possess dangerous infective qualities, its deficiency in nitrogenous elements, fat and sugar, and the increased proportion of earthy salts, would render it objectionable as an article of diet. It has long been known that it was liable to produce diarrhœa and debility in infants; but though many children fed on such milk have died from general or localized tuberculosis, the part probably played by this food in its production has not been suspected. One of the first matters to be taken into consideration at this point is a reconsideration of the question whether bovine tuberculosis is essentially the same disease in kind as the human form.

Now, it may be said at the outset that it differs in its morphology and also in its mode of development. The tuberculosis of man tends to become caseous, in cattle to be cretaceous. The cretaceous change follows the caseous in the human family, and is a rarity. The caseous change is a rarity in animals. Ulcers and cavities (vomicæ) are characteristic appearances in the human disease; they occur rarely, if at all, in the animal variety.

The little centres of disease grow by the deposit of cretaceous matter in concentric layers until the pulmonary lobule is distended to many times its normal size, so that at length in some cases it bursts through the boundaries of the lobule and unites with a similarly extending deposit in an adjacent lobule, the two forming a yellow tumor of stony hardness. Meanwhile the inter-lobular tissue is filled with little tumors, usually pedunculated, that vary much in size, each developing a yellow centre. The visceral and parietal layers of the pleura are also covered with similar tumors, which are then known by the name of "grapes," 'angleberries," etc. Now the affected glands develop in a similar way in cattle by "concretive accretion," while in the human kinds they first atrophy before the deposit of lime salts takes place. Virchow,* accordingly, has been inclined to regard the changes as similar to those that attend the development of tumors, and prefers to consider them in the category of the lympho-sarcomata. At this point it is but proper to advert for a moment to the remarkable views of Dr. Charles Creighton,† of Cambridge, England. This observer, in conducting post-mortem examinations on phthisical subjects, was led to think that a certain class could be separated from the ordinary varieties, and these presented certain peculiarities similar to those described in the pearl disease (bovine tuberculosis, *Merlinsigkelt*, *Duckweed*, *Potato Disease*). In the twelve

* *Virchow.* The Monthly Rec. of Med. and Pharm. Feb., 1882.

† *Creighton.* Bovine Tuberculosis in Man. London, 1881.

cases which serve to form the basis of his views, he lays stress upon certain (1) leaf-like or cord-like outgrowths of the pleura and peritoneum; (2) distinctly nodular formations in the lymphatic glands; and (3) the smooth-walled vomicæ or encapsuled nodules. Apropos of these points, it may be said here (1) that the writer's experience in two cases of bovine tuberculosis do not lead him to believe that the "leaf-like or cord-like outgrowths" are distinctive of the bovine disease, and that there is a danger of mistaking cross-sections of terminal bronchial twigs for smooth-walled vomicæ; a criticism which has already been made by one who has authority.

Returning to the danger of infection which some writers claim, it is well to hear from Toussaint,* whose name has latterly been associated with that of Pasteur in the field of pathological investigation. He says:

"I have studied tuberculosis, in its different modes of infection, and I am able to say, after having performed a very long series of experiments upon hogs, rabbits and cats, that no other *contagious disease possesses greater virulence.* The inoculation of a rabbit is followed by results as positive as those obtained from charbon; the same proved true of the other varieties of animals experimented upon.

"In tuberculosis all the fluids of the economy, the nasal mucus, the saliva, the serosity of the tissues and the urine, are virulent and can communicate the disease. Respecting the virus itself, it resists and preserves its action at the temperature which *destroys the bacteria of charbon.*

"If in the human race tuberculosis appears to be less malignant, it is because it often manifests itself in a slow, chronic form, which may last for years, and even sometimes be susceptible of cure; it is, however, none the less redoubtable, and the profession is aware that such cases are so exceptional that they may be counted. Contagion is also very difficult to ascertain, because of the slow appearance of the phenomena.

"The following experiments demonstrate the *resistance of the virus and the danger there is in the employment of meat and the debris of tuberculous animals.*

"By means of a press, I have extracted from the lungs of a cow affected with tuberculosis, presenting an œdema of the anterior lobe,

---

* *Toussaint.* Monthly Record of Pharm., 1882. (Translation.)

quite a large quantity of juice, containing little virus, almost transparent. Of this I injected 15 grm. under the skin of the inferior portion of the ear of a pig and 10 drops into two rabbits.

"Then I injected the same quantities of this liquid, heated in a water bath from 55 degs. to 58 degs. (Cent.) for ten minutes, in the same region of four hogs and four rabbits.

"These animals having been completely isolated from each other, were kept under observation. I very readily ascertained the development and ordinary march of the disease: local tubercle and hard engorgement of the parotid ganglions.

"General infection occurred very rapidly in every one of these animals; curiously to state, the rabbits which had been inoculated with the heated liquid died before the others.

"One of the hogs was killed two months after the injection had been made; the autopsy revealed a local caseous tubercle, an enormous parotid ganglion, containing at that early date cretaceous points. In the lung a large number of gray granulations were found, and also tubercles in the spleen and liver.

"After the third month a second hog was killed, and at the same time the pig which had been inoculated with the liquid that had not been submitted to the heat. The difference between the lesions of these two animals was very slight; the condition, however, of the latter was more advanced.

"The pulmonary tubercles of the hogs which had been inoculated with the heated juice were afterwards injected into rabbits, which, in their turn, became tuberculous. Two of these latter, killed at the end of the three months, gave evidences of numerous lesions in the lung, spleen, kidneys, and serous membranes.

"Two of the hogs which were inoculated with the heated liquid are still alive. Five months after the operation, however, one of them is almost dead.

"Of the four rabbits inoculated with the heated extract, one died accidently on the thirty-fifth day; the parotid ganglion was found to be caseous, but there was no general infection. The others died of generalized tuberculosis on the one hundred and sixty-fourth to the one hundred and seventieth day; one of them presented highly developed osseous lesions of the anterior members; there was a formation of caseous pus in the articulations of the shoulder and leg, the articular surfaces, and even a portion of the diaphyses were completely destroyed.

"Respecting the rabbits inoculated with the juice which had been heated, one was killed forty-three days after the injection. It presented numerous gray tubercles in the lung and liver. The second, a female, is still living; since its inoculation it has had three litters; of the first, the entire number died the day after their birth; the second consisted of five young, which have, as has also the last litter, been kept in order to ascertain if there is any hereditary taint. As the mother is at this moment affected with a very advanced tuberculosis, it will be interesting to ascertain the successive states through which they will pass.

"2 degs. *Slices of the flesh taken from the thigh of a tuberculous sow were placed upon a chafing-dish and exposed to gas heat;* they were cooked rare, afterwards they were submitted to pressure, and, with the juice obtained, two rabbits were inoculated; two others were injected with the liquid when cold. *These latter died on the twenty-fifth day, almost the same, of a caseous pneumonia and tubercles in all the tissues.*

"Of the two rabbits which were inoculated with the heated juice, one died on the fifty-sixth day, and we found local and ganglionic lesions, gray granulations in the lung, epiploon and the spleen; the other is still alive, but it has emaciated, and death will soon supervene."

Regarding the results of these experiments, it may be said at once that those in which rabbits were used could not be accepted as reliable, and that in any case the results obtained in the hogs did not necessarily indicate any special virulence for the tubercular matter, since it has been proved (Fox, Sanderson, Carter, and Andrew Clark*) that inoculation of any foul organic matter will set up processes that have the gross and minute appearance of tubercle. It is needless to say that these phenomena had previously been observed, and in no way militate for or against the theory of a specific virus in tubercle.

Virchow seems, therefore, to have been justified when he said,† recently, neither "experiments nor clinical observations, up to the present moment, have furnished any decisive facts. We should, there-

* Sir Andrew Clark has found that any kind of *pus* injected into rabbits will produce tubercle. Address at Bellevue Hospital Medical College, Dec. 14, 1878. Med. Rec.

† Mar. 10, 1880.

fore, consider the subject in a state of abeyance; for if one is desirous at present of arriving at a conclusion by comparing the positive and negative results that have been reached—number of positive results and those negative, especially in forgetting that the proportion of animals used for investigative purposes became spontaneously tuberculous is considerable—he must go beyond the facts in the case and abandon himself to his individual frame of mind. It is possible to think that hazard plays an important rôle in the results, and even if we are unwilling to attribute any part to it, nevertheless it is by no means established that we are not in the presence of a characteristic morbid entity of a specific virulence. For one, at least, who cannot recognize the similarity of the tubercles of the bovine race and the caseous products, the results obtained, so incongruous and contradictory, that the idea of positive specific virus does not appear to me to have been yet scientifically demonstrated. Therefore, in the light in which I consider it, experimental investigation should be devoted to ascertaining if, by any alimentation, consisting of different, altered and unwholesome substances, a formation of similar products will not occur in the organism of our domestic animals. Moreover, it is only by including in our researches a very large series of subjects and not isolated cases, as some physicians have done lately, that we will succeed in learning in what proportions pre-existing or coincident affections force the result of the conclusions."

To much the same effect is the report of Prof. Siedamgotzky,* employed by the chancellor of the King of Saxony to inquire "whether and how far the use of the meat and milk of cows which have the pearl disease is baneful to human beings." He reported that his experiments (conducted on hogs and sheep) yielded no result giving any positive support to the statement that tuberculosis can be conveyed to mankind through the milk or meat of tuberculous (pearly) cattle.

The evidence of Law on an allied point is also important. According to him (Nat. Bd. of Health Bull., 40, 1882) we have no satisfactory evidence that tuberculosis has ever been conveyed by bovine lymph. And yet he states that Jersey cattle, which he thinks are especially prone to tubercle, have preference in the selection; and besides, he adds, the cutaneous irritation of induced cow-pox will not

* *Siedamgotsky* Arch. f. Wiss. und pract. Thier-hlke, March 8, 188[illegible].

only enlarge the adjacent lymph glands, but leave them permanently so, and they will then manifest fever and other evidences of tuberculosis.

In closing this brief and necessarily incomplete review of a subject that has vast practical importance, the writer ventures to formulate certain conclusions that he has derived from his personal experience and a study of the literature of tuberculosis. It will have been observed that the progress made in our elucidation of the nature of tuberculosis has been accomplished mainly through the experiments of the past ten years in comparative pathology. We should, therefore, hope that these researches may be successfully continued until further results are reached which will have still greater practical value, and that we may at length be furnished with weapons for successfully resisting that dreaded disease—consumption.

The following are the conclusions:

1. Tuberculosis is a disease that is hereditary, statistics having abundantly proved that there resides in certain families a greater or less susceptibility to the disease, which usually manifests itself in the pulmonary organs. It generally attacks such individuals as are deficient in physical vigor, whether as the result of traumatism, continued illness, or congenital vice of constitution. It is apt to occur as a sequel to certain diseases, such as measles and whooping-cough, and is antagonized by others, such as empysema or some cardiac affections. Residents in great altitudes are almost exempt; while those who live in warm and moist and exceedingly variable climates are specially prone to it. It is no less true that the precautions taken by intelligent persons who have been in a position to withstand the predisposing causes of the disease, have often enabled them either to escape the disease altogether; or, if it has been contracted, appropriate treatment has enabled them to resist it successfully.

2. The most distinguishing characteristic of tubercle is the occurrence in the tissues, of minute, bright, glistening translucent particles, that have been called miliary tubercles, granula, granulations, etc.

3. They are the result of an inflammatory process, because they can be produced by the introduction of mechanical irritants into the system.

4. When these minute bodies coalesce to form larger bodies and undergo a change of color, they are known as crude or yellow tubercles.

5. Some of them contain the reticulated tissue that is called adenoid, and resembles most closely the framework of the lymphatic glands. As their age advances, the centre is quite apt to be occupied by a giant-cell, a large multi-nucleated body, whose boundaries and processes are hard to define, because they shade off gradually into the surrounding tissue. In some cases Schueppel's epithelial cells are found, but neither they nor giant-cells are essential elements of tubercle.

6. No region of the body appears to be exempt from them, though the lungs and serous membranes are most frequently known to be attacked.

7. In the gradual development of these bodies they undergo caseous change at the centre, which phenomenon is another marked feature of tubercle. Still, in some instances, we have reason to suppose that the miliary tubercle may become organized, and thus a cure result. There are cases where the tubercle does not exhibit the reticulated structure, but the fibrillated or fibrous, and in these instances it is conjectured that the most favorable issue is to be expected.

8. Tubercles rarely remain long in situ without setting up an additional inflammation, that may best be classed as a pneumonia. It is the *infiltrated tubercle* of Laennec, the *catarrhal pneumonia* of Niemeyer, or the *desquamative pneumonia* of Buhl. It may perhaps be protective in some instances, serving to wall off a caseous process, thus preventing it from becoming disseminated, or it may eventually itself participate in the same process and lead finally to necrosis of the lung and the production of cavities.

9. Tubercles may be confined to a limited area and a single lobe of the lung, or a single lung, or they may be diffused pretty equally in different organs; generalized, disseminated, or secondary tuberculosis is the most dangerous and malignant, and is due to transmission of the disease by the lymphatics or blood vessels, usually the latter. In this secondary form the first manifestations are the gray granulations, as they are also in the primary form.

10. It is not yet certain whether the artificial or false tubercle, produced by the inoculation of non-tuberculous matter, is a local disease merely, incapable of being generalized.

11. Tuberculosis is inoculable and it "breeds true," always producing its kind if it produces anything, but other substances will also in a certain number of cases produce the same results; in fact, any organic substance that is capable of physical deterioration. It has not been satisfactorily proved that tuberculosis has a specific virus.

12. There is some good evidence going to prove that tubercle is contagious, *i.e.*, that it is capable of propagation by cohabitation, or, in other words, close association with persons that have the disease. The number of well-authenticated instances in the human being where the disease appears to have been spread in this way indicate something more than a coincidence This relation has been shown with a reasonable amount of certainty in horned cattle.

13. And yet bovine tuberculosis differs in some important particulars from the disease in men and many of the lower animals, though it is highly probable that the differences, which are mainly in the pathological appearances, are referable to differences of anatomical structure in the different animals.

14. The evidences that Creighton has brought forward to show that there is in man a special form of tuberculosis similar to that in horned cattle requires further substantiation.

15. From a sanitary standpoint we should discountenance the use of milk or meat from phthisical cows, since if not absolutely infective, they are at least deficient in the proper nutritive elements, and for this reason alone should be excluded from the markets.

16. Though it does not appear that it can be shown that any person has ever been rendered tuberculous through the medium of bovine virus, still when the cattle used for vaccine purposes are slaughtered, those in charge of the farms should examine the viscera of all such cattle before the virus is furnished to physicians, and it should be specially understood that such an examination has been made in every instance where the virus is sold.

17. The parasititic theories of Klebs, Koch and others are still *sub judice.*

